# HemaMax™, a Recombinant Human Interleukin-12, Is a Potent Mitigator of Acute Radiation Injury in Mice and Non-Human Primates

**DOI:** 10.1371/journal.pone.0030434

**Published:** 2012-02-24

**Authors:** Lena A. Basile, Dolph Ellefson, Zoya Gluzman-Poltorak, Katiana Junes-Gill, Vernon Mar, Sarita Mendonca, Joseph D. Miller, Jamie Tom, Alice Trinh, Timothy K. Gallaher

**Affiliations:** 1 Neumedicines, Inc., Pasadena, California, United States of America; 2 Department of Cell and Neurobiology, Keck School of Medicine, University of Southern California, Los Angeles, California, United States of America; University of Pittsburgh, United States of America

## Abstract

HemaMax, a recombinant human interleukin-12 (IL-12), is under development to address an unmet medical need for effective treatments against acute radiation syndrome due to radiological terrorism or accident when administered at least 24 hours after radiation exposure. This study investigated pharmacokinetics, pharmacodynamics, and efficacy of m-HemaMax (recombinant murine IL-12), and HemaMax to increase survival after total body irradiation (TBI) in mice and rhesus monkeys, respectively, with no supportive care. In mice, m-HemaMax at an optimal 20 ng/mouse dose significantly increased percent survival and survival time when administered 24 hours after TBI between 8–9 Gy (p<0.05 Pearson's chi-square test). This survival benefit was accompanied by increases in plasma interferon-γ (IFN-γ) and erythropoietin levels, recovery of femoral bone hematopoiesis characterized with the presence of IL-12 receptor β2 subunit–expressing myeloid progenitors, megakaryocytes, and osteoblasts. Mitigation of jejunal radiation damage was also examined. At allometrically equivalent doses, HemaMax showed similar pharmacokinetics in rhesus monkeys compared to m-HemaMax in mice, but more robustly increased plasma IFN-γ levels. HemaMax also increased plasma erythropoietin, IL-15, IL-18, and neopterin levels. At non-human primate doses pharmacologically equivalent to murine doses, HemaMax (100 ng/Kg and 250 ng/Kg) administered at 24 hours after TBI (6.7 Gy/LD_50/30_) significantly increased percent survival of HemaMax groups compared to vehicle (p<0.05 Pearson's chi-square test). This survival benefit was accompanied by a significantly higher leukocyte (neutrophils and lymphocytes), thrombocyte, and reticulocyte counts during nadir (days 12–14) and significantly less weight loss at day 12 compared to vehicle. These findings indicate successful interspecies dose conversion and provide proof of concept that HemaMax increases survival in irradiated rhesus monkeys by promoting hematopoiesis and recovery of immune functions and possibly gastrointestinal functions, likely through a network of interactions involving dendritic cells, osteoblasts, and soluble factors such as IL-12, IFN-γ, and cytoprotectant erythropoietin.

## Introduction

Use of ionizing radiation or nuclear devices as weapons of terrorism is now recognized as a major public health threat. In the event of a nuclear detonation, terrorist radiological (e.g., “dirty”) bomb, or attack on a nuclear power plant in a populated area, mass casualties will occur that will be in the need of immediate medical attention [1]. At exposures approximating 4 Gy, it is estimated that 50% of individuals will die within 60 days unless there is medical intervention [2]. The majority of deaths that occur from exposures of 2–10 Gy will result from the combined effects of immune, hematopoietic, and gastrointestinal (GI) failure, as these are the most radiosensitive tissues [1]–[3]. To date, there are no FDA approved therapeutic agents capable of increasing the chance for survival by simultaneously promoting or accelerating the recovery of the immune, hematopoietic and gastrointestinal compartments following radiation injury.

In the event of a radiation disaster or act of terrorism affecting a large civilian population, the goal would be to provide a potent frontline therapy that increases the chance for survival of the exposed, or potentially exposed, individuals. One of the challenges in such events is that medical care and treatments will not be available immediately following radiation exposure. It is envisioned that it will take 24 hours or more to mobilize medical teams and necessary life-saving drugs and equipment to the scene of a radiation disaster [4].

Since medical care will not be immediately available, a medical intervention capable of increasing the chance for survival as a frontline therapy would have to be efficacious when administered at protracted time points following radiation exposure. This is indeed a challenge in that total body irradiation (TBI) causes massive apoptosis to rapidly dividing cells in radiosensitive organs, such as the peripheral blood, bone marrow, and GI tract, starting immediately after radiation exposure [1], [2]. Moreover, the chance of successfully providing life-saving treatment to the exposed individuals decreases exponentially following radiation injury. Thus, the effectiveness of providing countermeasure treatments that could alleviate damage caused by radiation decreases rapidly with time.

Given this challenge, there has been a search for radiomitigation drugs that can increase the chance for survival following radiation exposure to sensitive tissues such as the immune system, bone marrow, and GI tract Numerous cytokines proteins and small molecules,are under evaluation to to assess their respective radiomitigation potential . For example Johnson et al [5] report on a small molecule cyclin-dependent kinase inhibitor which shows radiomitigation in mice when administered 20 hours after irradiation. Protein drugs based upon the bacterial flagellin protein have been shown to be radioprotective at administration prior to radiation [6] or up to four hours post-irradiation [7], but not at 24 hours post-irradiation [7]. Recombinant human growth hormone has been reported to confer increased survival in mice when treated for five consecutive days when administered first up to 12 hours after irradiation [8]. Reviews by Singh and Yadav [9], Hérodin and Drouet [10], Weiss and Landauer [11], and Dumont et al [12] provide further information on radioprotectants and mitigants.. To date, there are no reports of any of these agents conferring increased survival at 24 hours or longer post radiation exposure, either in mice or non-human primates.

HemaMax is a recombinant human interleukin-12 (IL-12) preparation that is currently being developed for use against acute radiation syndrome in humans. Previously, we have investigated the radioprotective properties of a prototype preparation, m-HemaMax, a recombinant mouse IL-12, following intravenous administration. We found that m-HemaMax was able to dramatically increase survival in mice following exposure to lethal doses of TBI when it was administered at a single, low dose, either 24 hours before or within 1 hour after radiation exposure [13], [14]. Moreover, multilineage recovery of peripheral blood cell counts via stimulation by m-HemaMax, namely white blood cells, red blood cells and platelets, was observed in both normal and tumor-bearing mice exposed to sublethal TBI [13], [14]. In these radioprotection studies, m-HemaMax showed a remarkable ability to reconstitute the bone marrow compartment following ablation [13], [14]. Overall, these data suggested that the activity of m-HemaMax in these model systems is initiated at the level of primitive cells, likely hematopoietic and non-hematopoietic stem cells, residing in the bone marrow compartment, and that activation of these primitive cells leads to regeneration of the bone marrow compartment following radioablation or radiosuppression [13], [14].

IL-12 is a heterodimeric cytokine, comprising both p40 and p35 subunits, that is well-known for its role in immunity [15]. In numerous reports spanning about two decades, IL-12 has been shown to have an essential role in the interaction between the innate and adaptive arms of immunity by regulating inflammatory responses, innate resistance to infection, and adaptive immunity [15]. Endogenous IL-12 is required for resistance to many pathogens and to transplantable and chemically induced tumors. The hallmark effect of IL-12 in immunity is its ability to stimulate the production of interferon-gamma (IFN-γ) from natural killer (NK) cells, macrophages and T cells [15]. Further, several *in vitro* studies in the early-mid nineties reported that IL-12 is capable of stimulating hematopoiesis synergistically with other cytokines [16]–[20]. The hematopoiesis-promoting activity of IL-12 appears to be due to a direct action on bone marrow stem cells as these studies used highly purified progenitors or even single cells. The role of IFN-γ in the hematopoietic activity of IL-12 is not clear as several studies have linked both the promotion and suppression of hematopoiesis to IFN-γ [21]–[27]. The potent effect of IL-12 in mitigating radiation injury following exposure to lethal doses of radiation was previously unknown.

Since we previously found that m-HemaMax was highly effective when administered shortly after TBI, we attempted to utilize a more stringent model of radiation exposure to investigate the ability of m-HemaMax and HemaMax to increase survival when administered at protracted time points post radiation in mice and non-human primates (NHP), respectively. In a model of radiomitigation, where single, low doses of m-HemaMax in mice or HemaMax in NHP are administered subcutaneously at 24 hours or longer post irradiation, we now show that HemaMax can provide potent mitigation of radiation injury to multiple tissues, including the immune, bone marrow, and GI compartments, leading to significant increases in survival. These results are reported herein for both murine and NHP radiomitigation models in the complete absence of supportive care. To our knowledge, this is the first report showing potent radiomitigation effects of a therapeutic agent in mice and NHP at protracted time points post radiation, such as 24 hours or longer, following acute ionizing radiation exposure.

## Materials and Methods

### Ethics Statement

Mice studies were carried out in BATTS Laboratories (Northridge, CA, USA; Animal Welfare Assurance Number (AWAN) from the Office of Laboratory Animal Welfare (OLAW): A4475-01), the Roy E. Coats Research Laboratories (University of California, Los Angeles, CA, USA; AWAN from OLAW: A3196-01), or LAB Research, Inc. (Laval, Québec, Canada; AWAN from OLAW: A5525-01). All procedures were reviewed and approved by the Institutional Animal Care and Use Committees (IACUC) of BATTS Laboratories (permit number: 01012011), Roy E. Coats Research Laboratories, and LAB Research, Inc., (permit number: 2009-2665). Each of these institutions is accredited by the Association for the Assessment and Accreditation of Laboratory Animal Care (AAALAC) and the American Association of Laboratory Animal Care. During the study, care and use of animals were conducted in accordance with principles outlined in the Guide for the Care and Use of Laboratory Animals published by the US National Institutes of Health (publication No: 85-23, revised 1996) and the Guide to the Care and Use of Experimental Animals published by the Canadian Council on Animal Care.

Care and use of NHP were in accordance with principles outlined in the current Guide to the Care and Use of Experimental Animals published by the Canadian Council on Animal Care, the Guide for the Care and Use of Laboratory Animals published by the Institute of Laboratory Animal Resources, and the recommendations of the Weatherall report for The Use of Non-Human Primates in Research (December 2006). All procedures for NHP studies were reviewed and approved by the IACUC of LAB Research, Inc., (permit numbers: 2009-1243 and 2009-1253). The NHP irradiation was approved by the Canadian Nuclear Safety Committee (radiation permit number: 3572637). The LAB Research, Inc., is an OLAW assured and AAALAC accredited facility. During the study, all efforts were made to minimize suffering.

### m-HemaMax and HemaMax

m-HemaMax (recombinant murine IL-12) was purchased from Peprotech (Rocky Hill, NJ, USA) or provided by SBH Sciences (Natick, MA, USA) exclusively to Neumedicines. HemaMax (recombinant human IL-12) was provided by SBH Sciences (Natick, MA, USA) exclusively to Neumedicines. In the initial mouse survival studies, lyophilized m-HemaMax was dissolved in phosphate buffer saline (PBS), pH = 7.2. In all other studies, m-HemaMax and HemaMax were dissolved in a proprietary formulation containing trehalose, Tween 20, and sodium phosphate, pH = 5.6 (P5.6TT). Studies in mice and rhesus monkeys utilized m-HemaMax and HemaMax, respectively. PBS was used as vehicle in the initial mouse survival studies as indicated. P5.6TT was used as vehicle in all other studies.

### Mice

Mouse survival studies were carried out at either BATTS Laboratories (Northridge, CA, USA; HHS OLAW A4475-01) or the Roy E. Coats Research Laboratories (University of California, Los Angeles, CA, USA; HHS OLAW A3196-01). Mouse bone marrow isolations were carried out at BATTS Laboratories. Female C57BL/6 mice were obtained from The Jackson Laboratory (Sacramento, CA, USA), and male mice from Harlan Laboratories (Placentia, CA, USA), or were bred at the Roy E. Coats Research Laboratories (Coats mice) Coats mice are gnobiotic, and consequently,are have a lesser less radiosensitive than the Harlan mice. Differences in radiation doses in experiments using the different mice consequently differed with higher radiation amounts used in the Coats mice experiments. Coats mice exposed to radiation doses of 8.6, 8.8 and 9.0 Gy in these studies whereas Harlan mice were subjected to 8 Gy unless otherwise specified. Mouse pharmacokinetic (PK) and pharmacodynamic (PD) studies and gastrointestinal (GI) tissue isolations were carried out at LAB Research, Inc. (Laval, Québec, Canada; HHS OLAW A5525-01). Male C57BL/6 mice were obtained from Charles River Canada, Inc. (Saint-Constant, Québec, Canada). In PK/PD studies involving radiation Charles River mice were subjected to 8.6 Gy TBI (LD_100/30_). At all study sites, mice were maintained in quarantine for at least one week. Mice used in the survival and PK/PD studies were 9 weeks to 10 weeks old and weighed approximately 20 g with no signs of disease.

### Survival Studies in Mice

At day 0, TBI was carried out at a lethal dose of 8.0 Gy (Harlan mice) or 9.0 Gy (Coats mice) –doses that are expected to cause death in about 90% of animals within 30 days–using Gammacell® 40 with ^137^Cs source (Theratronics, Ontario Canada with a rate of 71 cGy/min in Coats mice studies and 85 cGy/min in the Harlan mice studies) in a specially constructed “pie-box” designed to keep mice in the center of the irradiator for even distribution of radiation. Mice received subcutaneous injections of either vehicle or m-HemaMax at the indicated doses at 24 hours, 48 hours, and/or 72 hours after irradiation. Mice were monitored for survival up to day 30. During this period, mice were deprived of all supportive care, including antibiotics, to increase the stringency of the survival protocol. The mice had access to food and acidified water *ad libitum*.

Radiation dose dependency of the m-HemaMax effect was evaluated in mice (n = 10 per group; Coats mice), which were irradiated at lethal doses of 8.6 Gy, 8.8 Gy, and 9.0 Gy, which resulted in LD_70/30_, LD_90/30_, and LD_100/30_, respectively. Animals received vehicle or m-HemaMax at a dose of 20 ng/mouse 24 hours after TBI. Mice were monitored for survival up to day 30. No supportive care, including antibiotics, was allowed during this period. The mice had access to food and acidified water *ad libitum*.

### Plasma PK and PD of m-HemaMax in Irradiated and Non-Irradiated Mice

Mice (n = 3 per group) received m-HemaMax subcutaneously at a dose of 10 ng/mouse, 20 ng/mouse, 40 ng/mouse, or 200 ng/mouse either in the absence of irradiation or 24 hours after an LD_100/30_ (8.6 Gy; Charles River mice) of TBI. Two additional control groups of animals (n = 3 per group), which did not receive m-HemaMax, were either not exposed to radiation or irradiated at 8.6 Gy. The concentrations of m-HemaMax and IFN-γ were determined in plasma from blood samples withdrawn at 45 minutes and 1.5, 3, 6, 12, 24, 48 and 72 hours after m-HemaMax administration by enzyme-linked immunosorbent assay (ELISA). Plasma erythropoietin (EPO) levels were measured only at the 12 hour timepoint because of limited sample availability.

### Bone Marrow and GI Histopathology in Mice

For bone marrow histopathology studies, mice (n = 2 per group) were subjected to TBI at 8.0 Gy (Harlan mice, ∼LD_40/30_ in this experiment) and were subsequently administered either vehicle (P5.6TT) or m-HemaMax (20 ng/mouse) subcutaneously at either (a) 24 hours, (b) 24 hours and 2 days, (c) 24 hours and 3 days, (d) 24 hours and 4 days, or (e) 24 hours and 5 days after irradiation. An additional group of mice (n = 2) received HemaMax at 24 hours after TBI. Mice were sacrificed 12 days after irradiation, and femoral bone marrow was provided as paraffin-embedded, sectioned tissues by Cyto-Pathology Diagnostic Center, Inc (Duarte, CA, USA).

For GI histopathology studies, mice (n = 3 per group) received vehicle (P5.6TT) or m-HemaMax subcutaneously at doses from 10 ng/mouse to 200 ng/mouse either in the absence of irradiation or 24 hours after a TBI at 8.6 Gy (Charles River mice, LD_100/30_). Mice were sacrificed 3 days after irradiation, and jejunum was provided as paraffin-embedded, sectioned tissues by Cytopathology Diagnostics Center, Inc. (Duarte, CA, USA).

Sectioned tissues were deparaffinized with xylene, rehydrated with decreasing concentrations of ethanol, and subjected to the heat-induced epitope retrieval (HIER) to recover antigens. Endogenous peroxidase was inhibited with 0.3% H_2_O_2_, and background staining was blocked with the Background Sniper (Biocare Medical, LLC.; Concord, CA).

In the bone marrow histopathology studies, tissue sections were incubated with either rabbit anti-mouse IL-12 receptor beta 2 subunit (IL-12Rβ2) (Sigma; St Louis, MO), rabbit anti-mouse osteocalcin (Millipore; Billerica, MA), a marker of osteoblasts, or rabbit anti-mouse Sca-1 (Epitomics; Burlingame, CA), a marker of hematopoietic stem cells. In the GI histopathology studies, tissue sections were incubated with rabbit anti-mouse IL-12Rβ2, or rabbit anti-mouse leucine-rich-repeat-containing G-protein-coupled receptor 5 (LGR5), a GI stem cell marker that is expressed upon GI injury. After removing the primary antibodies, tissue sections were incubated with peroxidase conjugated anti-rabbit IgG (ImmPRESS; Vector Laboratories; Burlingame, CA). Red coloring of peroxidase labeled cells developed following incubation with AEC substrate (ImmPACT AEC; Vector Laboratories; Burlingame, CA) and were counterstained with CAT Hematoxylin (Biocare Medical, Concord, CA). Tissue sections were then immersed in Vectamount (Vector Laboratories; Burlingame, CA), covered with a cover slip, sealed with clear nail polish, and visualized using an Olympus Compound microscope (Olympus America, Inc; Center Valley, PA) at 100× magnification for bone marrow sections and 400× for jejunum.

Co-expression of Sca-1 and IL-12Rβ2 on hematopoietic stem cells was evaluated by incubating bone marrow tissue sections first with rabbit anti-mouse Sca-1 (Epitomics, Burlingame, CA) followed by incubation with Rabbit on Rodent HRP-Polymer (Biocare Medical; Concord, CA) and 3,3′-diaminodbenzidine substrate (Biocare Medical; Concord, CA). After treatment with denaturing solution (Biocare Medical; Concord, CA), tissue sections were incubated with rabbit anti-mouse IL-12Rβ2 (Sigma; St Louis, MO) followed by incubation with Rabbit on Rodent AP polymer (Biocare Medical; Concord, CA) and Warp Red substrate (Biocare Medical; Concord, CA). Tissue sections were then counterstained in CAT Hematoxylin and visualized as described above. Using this method, cells expressing Sca-1 and IL-12Rβ2 were stained in brown and pink, respectively.

### Non-Human Primates (NHP)

Male rhesus monkeys, *Macaca mulatta*, were purchased from Worldwide Primates, Inc., (Miami, FL, USA). Animals of 3 to 4 years of age weighing 3.5 to 5.8 Kg were acclimatized for at least 7 weeks. All rhesus monkeys included in the experiments were in good health by physical examination, were negative for Herpes B-virus, simian immunodeficiency virus, simian T-lymphotropic virus, and simian type retrovirus, and were vaccinated against hepatitis A and measles. Animals were housed individually in stainless steel monkey cages equipped with automatic watering systems. The animal room environment was continuously controlled for temperature (21±3°C), humidity (30% to 70%), light cycle (12 hours light∶12 hours dark), and air change (10 to 15 air changes/hour). A standard certified commercial primate chow was available to each monkey twice a day. Food was withdrawn overnight prior to irradiation and necropsy. Animals were acclimated to the various procedures with positive reinforcement prior to study initiation. Health status was extensively evaluated to ensure animals were in good condition for the studies. All animals were provided prophylactic analgesia (buprenorphine) from day 5 to study completion. Specific euthanasia criteria were included in each experimental protocol to minimize suffering. Continuous clinical care (24 hours/7 days) were provided throughout the study to ensure prompt intervention when needed. A team of technicians and veterinarians trained in NHP medicine was responsible for clinical monitoring and provided state-of-the-art medical care.

### Allometric Dose Conversion From Mice to Rhesus Monkey

m-HemaMax doses that were found effective against lethal TBI in mice were converted to their equivalent doses in rhesus monkey based on body surface area [28]. Pharmacological equivalency of the species-specific equivalent doses were evaluated in relation to the HemaMax stimulation of IFN-γ secretion from peripheral blood mononuclear cells (PBMC) in vitro and the PK and PD characteristics of HemaMax in vivo.

### Isolation of CD14- PBMC and Quantification of IFN-γ Secretion

Human PBMC collected by apheresis were purchased from AllCells (Emeryville, CA, USA). Mouse and rhesus monkey PBMC were from Bioreclamation (Liverpool, NY, USA). CD14- PBMC were isolated as follows. Red blood cells were removed from human PBMC by a single step gradient with Ficoll-Hypaque premium (Density = 1.077; GE Healthcare Lifesciences; Piscataway, NJ, USA) and from rhesus monkey and mouse PBMC by lysis using ACK lysis buffer (Invitrogen; Carlsbad, CA, USA). To remove IL-12-secreting endogenous monocyte populations, human and rhesus monkey PBMCs were labeled with mouse anti-human CD14PE antibody (AbD Serotec; Raleigh, NC, USA), and mouse PBMCs were labeled with mouse anti-mouse CD14PE (AbD Serotec; Raleigh, NC, USA). The excess antibody was removed and cells were incubated with magnetic beads conjugated with anti-PE antibody (Miltenyi Biotec; Auburn, CA, USA). After removing the excess antibody, CD14+ cells were captured by adsorption to an LD column (Miltenyi Biotec; Auburn, CA, USA) immobilized in a magnetic field (Quadro MACS®; Miltenyi Biotec; Auburn, CA, USA). CD14- cells in the flow-through were collected, and those from humans were resuspended at a density of 14×10^6^ cells/mL in cold fetal bovine serum (FBS) containing 20% dimethyl sulfoxide whereas those from rhesus monkey and mouse were resuspended at a density of 2.14×10^6^ cells/mL in RPMI medium containing 10% FBS and antibiotics. IFN-γ was quantified by ELISA in supernatants from 2.5×10^5^ human, rhesus monkey, or mouse CD14- PBMC incubated with various concentrations (range: 0 to 1000 pM) of HemaMax or m-HemaMax for 16 hours at 37°C. All experiments were carried out in triplicate. The half maximal effective concentration (EC_50_) of IL-12 for stimulating IFN-γ secretion was calculated by SoftMax Pro® software version 3.1 (Molecular Devices; Sunnyvale, CA, USA) using a 4-parameter logistic fit.

### Plasma PK and PD of HemaMax in NHP

Radiation-naive rhesus monkeys received HemaMax subcutaneously at a dose of either 250 ng/Kg (n = 3) or 1000 ng/Kg (n = 3). The concentrations of HemaMax, IFN-γ, and other potential biomarkers of HemaMax were determined by ELISA in plasma samples withdrawn prior to the HemaMax administration and at 2, 6, 12, 18, 24, 30, 36, 48, 72, 96, 120, 144 and 168 hours after HemaMax administration.

### IL-12Rβ2 Expression in NHP and Human Bone Marrow and Small Intestine

Paraffin-embedded, sectioned tissues from NHP and human femoral bone marrow and jejunum/ileum were obtained from Biomax, Inc (Rockville, MD). NHP and human tissue sections were immunohistochemically stained for IL-12Rβ2 using rabbit anti-human IL-12Rβ2 according to the procedures described in the section for mice histopathology studies.

### Survival Studies in NHP

At day 0, rhesus monkeys acclimated to the restraining procedure with positive reinforcement were subjected to TBI at an LD_50/30_ of 6.7 Gy. Irradiation was performed in two half-dose fractions (anteroposterior and posteroanterior) at the rate of 55 cGy/minute using a Cobalt-60 unit (Theratron 780; Theratronics; Ontario, Canada). The irradiation dose was monitored with 2 dosimeters (Thermoluminescent or NanoDot dosimeters; Landauer Inc.; Glenwood, IL, USA) placed at the apex of the sternum and at the corresponding level in the interscapular area of each animal. Following TBI, animals were randomly assigned to receive subcutaneously either (a) vehicle at 24 hours post TBI (n = 8), (b) 100 ng/Kg of HemaMax at 24 hours post TBI (n = 8), (c) 100 ng/Kg of HemaMax at 24 hours and 7 days post TBI (n = 8), (d) 250 ng/Kg of HemaMax at 24 hours post TBI (n = 8), or (e) 250 ng/Kg of HemaMax at 24 hours and 7 days post TBI (n = 8). Animals were monitored for survival and clinical and physical characteristics for up to day 30. The primary outcome measure was the percentage of survival. Peripheral blood cell counts, body weight, and clinical signs were evaluated as secondary outcome measures.

During the study, blood transfusions or antibiotic use was prohibited. Evidence of pain or discomfort was treated with intramuscular buprenorphine (0.01 mg/Kg to 0.05 mg/Kg at least every 8 hours). Nutritive support (e.g. liquid diets) was provided if animals presented with decreased appetite. Throughout the study, clinical signs were monitored at least twice a day, and complete blood counts and body weight were monitored once every other day. Hematology samples were analyzed with an automated hematology analyzer (Advia 120; Bayer Diagnostics; Tarrytown, NY, USA). During the study, animals were euthanized if they had respiratory distress, anorexia/decreased appetite (complete anorexia for 3 days), weight loss (in excess of 20% of baseline body weight in 72 hours), unresponsiveness to touch, acute gross blood loss, generalized seizure, or abnormal vital signs. The euthanized animals or those found dead were subjected to a full macroscopic necropsy examination, including bacteriology testing. All animals were euthanized at the end of the study on day 31.

### Quantification of m-HemaMax and HemaMax and their Biomarkers in Plasma

Blood samples from mice and rhesus monkeys were collected into tubes containing ethylenediaminetetraacetic acid and were kept on ice (<30 minutes) until centrifugation. Samples were centrifuged at 1500× g for 10 minutes at 4°C. Plasma was aliquoted and stored at −70°C until use. Plasma m-HemaMax, HemaMax, and their potential biomarkers were assayed by ELISA. The ELISA kits for mouse IL-12 (p70) and IFN-γ were obtained from BioLegend (San Diego, CA, USA), for NHP IL-12 from BioLegend (San Diego, CA, USA), MabTech (Mariemont, OH, USA), and R&D Systems (Minneapolis, MN, USA), for NHP IFN-γ from MabTech (Mariemont, OH, USA), for human EPO, IL-18, and IL-15 from R&D Systems (Minneapolis, MN, USA), and for Neopterin from GenWay (San Diego, CA, USA). All assays were carried out in triplicate according to the manufacturers' instructions except those for NHP IL-12 in which an in-house reference standard was used instead of the standard provided by the manufacturer.

### Statistical Analyses

Data were presented as mean ± standard error (SE). Between-group differences in survival were evaluated with Kaplan-Meier survival analysis, followed by the Mantel-Cox Test for survival time and Pearson's chi-square test for percentage of survival. Between-group differences in blood cell counts were evaluated by analysis of variance (ANOVA), except for the number of platelet counts dropping below the transfusion level of 20,000 platelets/µl, which was analyzed by Pearson's chi-square test. Between group differences in clinical signs were evaluated by ANOVA. A *P* value of <.05 was defined as the level of statistical significance.

## Results

### Single, Low Doses of m-HemaMax Administered 24 Hours Post TBI Increased Survival in Irradiated Mice

**Figure 1:**
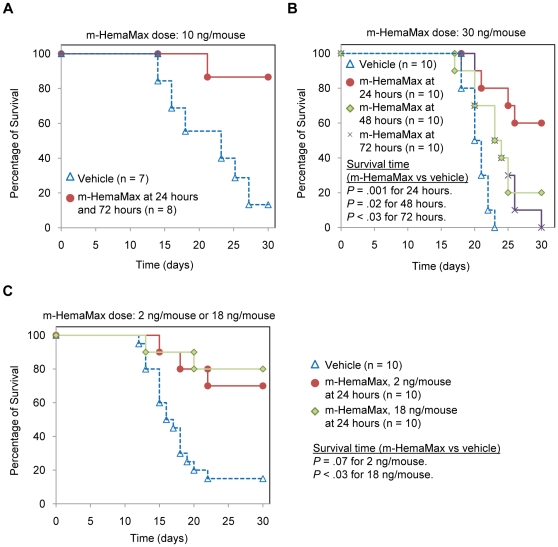
m-HemaMax administered at least 24 hours after TBI increased survival time of irradiated mice. (a) Animals received vehicle or m-HemaMax at an ostensible dose of 100 ng/mouse at 24 hours and 72 hours after a TBI of 8 Gy (LD_86/30_). (b) Animals received vehicle or a single, ostensible dose of 300 ng/mouse of m-HemaMax at 24 hours, 48 hours, or 72 hours after a TBI of 9 Gy (LD_100/30_). (c) Animals received vehicle or a single low dose of m-HemaMax (2 ng/mouse or 18 ng/mouse) at 24 hours after a TBI of 7.9 Gy (LD_85/30_). Vehicle and m-HemaMax were injected subcutaneously. Vehicle was PBS in (a) and (b) and P5.6TT in (c). The delivered m-HemaMax dose was estimated to be 10 ng/mouse in (a) and 30 ng/mouse in (b) because subsequent studies showed that the actual m-HemaMax dose delivered was approximately 10% of the intended dose, most likely due to m-HemaMax sticking to surfaces of vials and syringes.

In the initial studies, 87.5% of mice receiving a subcutaneous ostensible dose of 100 ng/mouse of m-HemaMax at 24 hours and 72 hours post 8 Gy TBI survived for up to 30 days, whereas only 14% of vehicle mice survived this same lethal 8 Gy TBI by day 30 (P<0.005) (Figure 1a). The actual m-HemaMax dose delivered in these studies was 10 ng/mouse (see below for a discussion concerning dose delivery). Subsequent studies evaluated whether a single dose of m-HemaMax was sufficient to provide similar radiomitigation effect. In these studies, a single, ostensible dose of m-HemaMax (300 ng/mouse; the actual delivered dose was 20–30 ng/mouse; see below for a discussion concerning dose delivery) significantly increased survival time when administered at either 24 hours (*P* = .001), 48 hours (*P* = .02), or 72 hours (*P*<.03 ) after a 9 Gy TBI resulting in the LD_100/30_ (Figure 1b). Mice treated with m-HemaMax had a higher percentage of survival when m-HemaMax was administered at 24 hours compared to 48 hours post TBI (Figure 1b). The difference in percentage of survival between the vehicle group and mice treated with m-HemaMax at 24 hours post TBI was statistically significant (0% vs 60%, respectively; *P*<.05) (Figure 1b).

It is noteworthy that in these early studies, very low, nanogram quantities of m-HemaMax were reconstituted in PBS. However, ELISA analysis of the reconstituted m-HemaMax demonstrated that the actual dose delivered was approximately 10% of the intended dose, most likely because of m-HemaMax sticking to surfaces of vials and syringes. Therefore, in the subsequent studies, m-HemaMax was reconstituted in P5.6TT, which increased dose delivery to nearly 90% of the intended dose. With this improvement, a single m-HemaMax dose of 2 ng/mouse or 18 ng/mouse provided significantly higher radiomitigation than did vehicle against a TBI dose of 7.9 Gy that resulted in an LD_85/30_ when administered 24 hours post radiation (Figure 1c). At the dose of 2 ng/mouse, m-HemaMax significantly increased percentage of survival (*P*<.02) and marginally increased survival time (*P* = .07) compared to vehicle. At the dose of 18 ng/mouse, m-HemaMax significantly increased both the percentage of survival (*P*<.005) and survival time (*P*<.03) compared to vehicle. Animals treated with m-HemaMax at a higher dose, such as 160 ng/mouse, had modestly longer survival time compared to the vehicle group but a lower percentage of survival relative to animals treated with the 2 ng/mouse or 18 ng/mouse dose (data not shown). Thus, these findings indicate that a dose of approximately 20 ng/mouse is the optimal, efficacious dose of m-HemaMax to increase survival.

**Figure 2:**
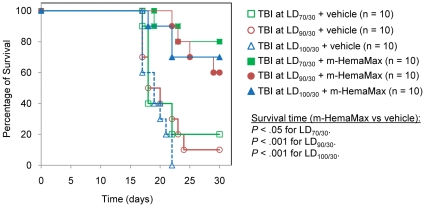
Efficacy of m-HemaMax in increasing survival is not dependent on radiation dose in mice. Animals were subjected to TBI at ascending radiation doses of 8.6 Gy (LD_70/30_), 8.8 Gy (LD_90/30_), and 9.0 Gy (LD_100/30_) and subsequently received m-HemaMax at a dose of 20 ng/mouse 24 hours after irradiation. Mice were monitored for survival up to day 30. Vehicle was P5.6TT.

To evaluate the relationship between the radiation dose and percentage of survival upon treatment with m-HemaMax, 3 ascending doses of radiation (8.6, 8.8, and 9.0 Gy corresponding to resultant LD _70/30_, LD _90/30_, and LD _100/30_, respectively) were tested in mice. m-HemaMax at a dose of 20 ng/mouse administered 24 hours after TBI significantly increased survival time at all 3 levels of radiation intensities (Figure 2). The percentage of survival in animals treated with vehicle was 20% at 8.6 Gy (LD_70/30_), 10% at 8.8 Gy (LD_90/30_), and 0% at 9.0 Gy (LD_100/30_) (Figure 2). Compared to the vehicle groups, treatment with m-HemaMax resulted in significantly higher percentage of survival of 80% at LD_70/30_, 60% at LD_90/30_, and 70% at LD_100/30_ (*P*<.05 for all) (Figure 2), demonstrating a radiation dose-independence for m-HemaMax administration at 24 hours post TBI within the selected window of radiation exposures. Remarkably, comparable percentages of survival after a single, fixed dose of m-HemaMax at increasing radiation doses indicate that the efficacy of m-HemaMax is not decreased with increasing radiation dose. These data suggest that at radiation doses where immune, bone marrow, and GI damage overlap, m-HemaMax can provide mitigation of injury in all three radiosensitive tissues, thereby leading to an increase in survival that is relatively independent of radiation dose within a certain window of exposure.

### Plasma PK and PD of m-HemaMax in Irradiated and Non-Irradiated Mice

**Figure 3:**
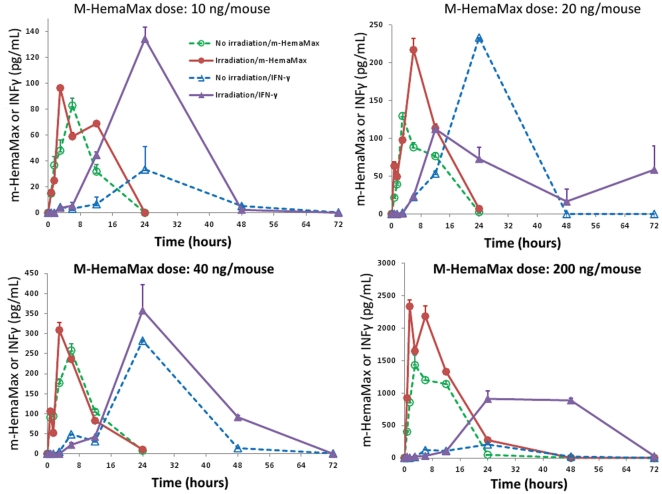
m-HemaMax administration increased plasma m-HemaMax and IFN-γ levels in irradiated and non-irradiated mice. Animals received m-HemaMax subcutaneously at a dose of (a) 10 ng/mouse, (b) 20 ng/mouse, (c) 40 ng/mouse, or (d) 200 ng/mouse in the absence of irradiation or at 24 hours after an LD_90/30_ of TBI. The plasma concentrations of m-HemaMax and IFN-γ were determined by ELISA in blood samples withdrawn at the indicated times. The y-axis scale in (d) is 8 times greater than those in (a) and (b) and 5 times greater than that in (c). n = 3 per timepoint in each group.

Plasma concentrations of m-HemaMax and IFN-γ were determined over 72 hours in 2 groups of mice, which received increasing doses of m-HemaMax (from 10 ng/mouse to 200 ng/mouse) either in the absence of irradiation or 24 hours after an approximate LD_90/30_ of TBI (8.6 Gy). The m-HemaMax doses lower than 10 ng/mouse were not evaluated because of the limitations in m-HemaMax detection. m-HemaMax was detected in all plasma from animals receiving m-HemaMax (Figure 3), but importantly, was not detectable in plasma samples from mice that did not receive m-HemaMax regardless of the presence or absence of irradiation (data not shown).

The exposure to m-HemaMax (area under the curve last; AUC_last_) increased dose proportionally from 10 ng/mouse to 40 ng/mouse regardless of the presence or absence of irradiation (Figure 3 and Table 1). Interestingly, maximum plasma concentrations (C_max_) of m-HemaMax were consistently higher in irradiated mice as compared to non-irradiated mice at all doses (Figure 3). The exposure to m-HemaMax (AUC_last_) at the dose of 200 ng/mouse was disproportionately higher than those at the lower doses (10 ng/mouse to 40 ng/mouse), suggesting that PK properties of m-HemaMax are non-linear at the higher dose ranges (Table 1). In the dose range of 10 ng/mouse to 40 ng/mouse, m-HemaMax reached C_max_ in 3 hours to 6 hours and was eliminated with a half-life of approximately 4 hours (Table 1).

**Table 1:** Plasma PK Characteristics of m-HemaMax in Irradiated and Non-Irradiated Mice.

	C_max_ (pg/mL)		AUC_last_ (pg.h/mL)		T_max_ (hours)		t_1/2_ (hours)	
m-HemaMax dose, ng/mouse	NR	R	NR	R	NR	R	NR	R
10	82.8	96.4	628	728	6	3	na	na
20	129.5	217.2	1453	2364	3	6	3.7	3.5
40	257.8	308.8	2720	2701	6	3	3.5	4.2
200	1428	2332	21008	37059	3	1.5	4.8	7.2

Animals received m-HemaMax subcutaneously at a dose of 10 ng/mouse, 20 ng/mouse, 40 ng/mouse, or 200 ng/mouse in the absence of irradiation or at 24 hours after an LD_90/30_ of TBI. The plasma concentrations of m-HemaMax were determined by ELISA.

AUC = area under the curve; C_max_ = maximum plasma concentrations; NR = no irradiation; R = irradiation; TBI = total body irradiation; T_max_ = time to achieve the maximum plasma concentration; t_1/2_ = half life.

m-HemaMax administration increased plasma IFN-γ concentration with a lag time at all study doses (Figure 3). Of significance, IFN-γ production was not abrogated in irradiated mice (Figure 3). In fact, for all m-HemaMax doses, except the optimal dose of 20 ng/mouse dose, plasma IFN-γ levels were higher in irradiated mice compared to non-irradiated mice (Figure 3). The exposure to IFN-γ dose proportionally increased as a function of increasing m-HemaMax dose from 10 ng/mouse to 200 ng/mouse (data not shown). Importantly, IFN-γ was not detected in plasma of mice, which did not receive m-HemaMax regardless of the presence or absence of irradiation.

**Figure 4:**
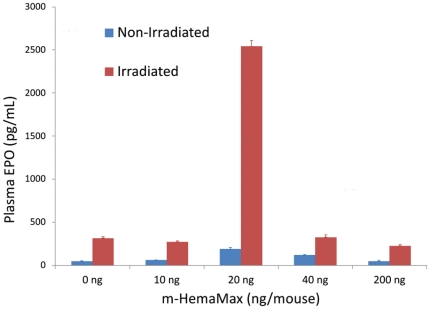
Optimal m-HemaMax dose of 20 ng/mouse increased plasma EPO concentration in irradiated mice. Animals received m-HemaMax subcutaneously at a dose of (a) 10 ng/mouse, (b) 20 ng/mouse, (c) 40 ng/mouse, or (d) 200 ng/mouse in the absence of irradiation or at 24 hours after an LD_90/30_ of TBI. The plasma concentrations of EPO were determined by ELISA in blood samples withdrawn at 12 hours after m-HemaMax administration.

Since preliminary studies had shown that co-administration of m-HemaMax and EPO in a certain regimen led to a substantial increase in survival following lethal radiation exposure (data not shown), we sought to assess whether m-HemaMax may affect plasma levels of EPO in irradiated and non-irradiated mice. Because of the limited sample availability, plasma EPO levels could be measured in only 1 early timepoint, 12 hours after m-HemaMax administration (Figure 4). In non-irradiated, untreated animals, EPO was detectable in plasma at low pg/mL range (Figure 4). Irradiation increased plasma EPO levels nearly linearly up to 80 hours post TBI, suggesting that EPO is a part of the physiological response to radiation injury (data not shown). Remarkably, however, at the optimal dose of 20 ng/mouse at 12 hours post administration (36 hours post radiation exposure), m-HemaMax substantially increased plasma EPO concentrations over the radiation-induced levels (Figure 4), indicating that m-HemaMax potentiates the EPO-mediated physiological response to radiation, but only at or near the optimal dosing level. It is noteworthy that, at this optimal dose, plasma EPO levels were also increased in non-irradiated mice (Figure 4). It remains to be further evaluated as to whether the EPO response to m-HemaMax administration occurs at a narrow window of m-HemaMax dose range because a highly potentiated EPO response was observed only after administration of the 20 ng/mouse dose (Figure 4). It is interesting to note that the IFN-γ response appeared to be subdued at the 20 ng/mouse dose of m-HemaMax, the dose at which EPO was upregulated by m-HemaMax, as compared to the other doses assessed. In a mice model of multiple sclerosis, administration of EPO was reported to downregulate the inflammatory response, and in particular, suppress IFN-γ [29]. Thus, these findings suggest that the increased plasma EPO levels may play a role in the suppression of plasma IFN-γ levels in irradiated mice that received m-HemaMax at the dose of 20 ng/mouse (Figure 3b), leading to a decrease in the inflammatory response to radiation.

Other biomarkers of m-HemaMax administration were also screened, namely tumor necrosis factor-alpha (TNF- α and stem cell factor (SCF), but the plasma levels for these factors were found to be below the limit of quantitation.

### Administration of m-HemaMax at 24 hours after TBI Mitigated Radiation-induced Injury in Murine Bone Marrow and Small Intestine

**Figure 5:**
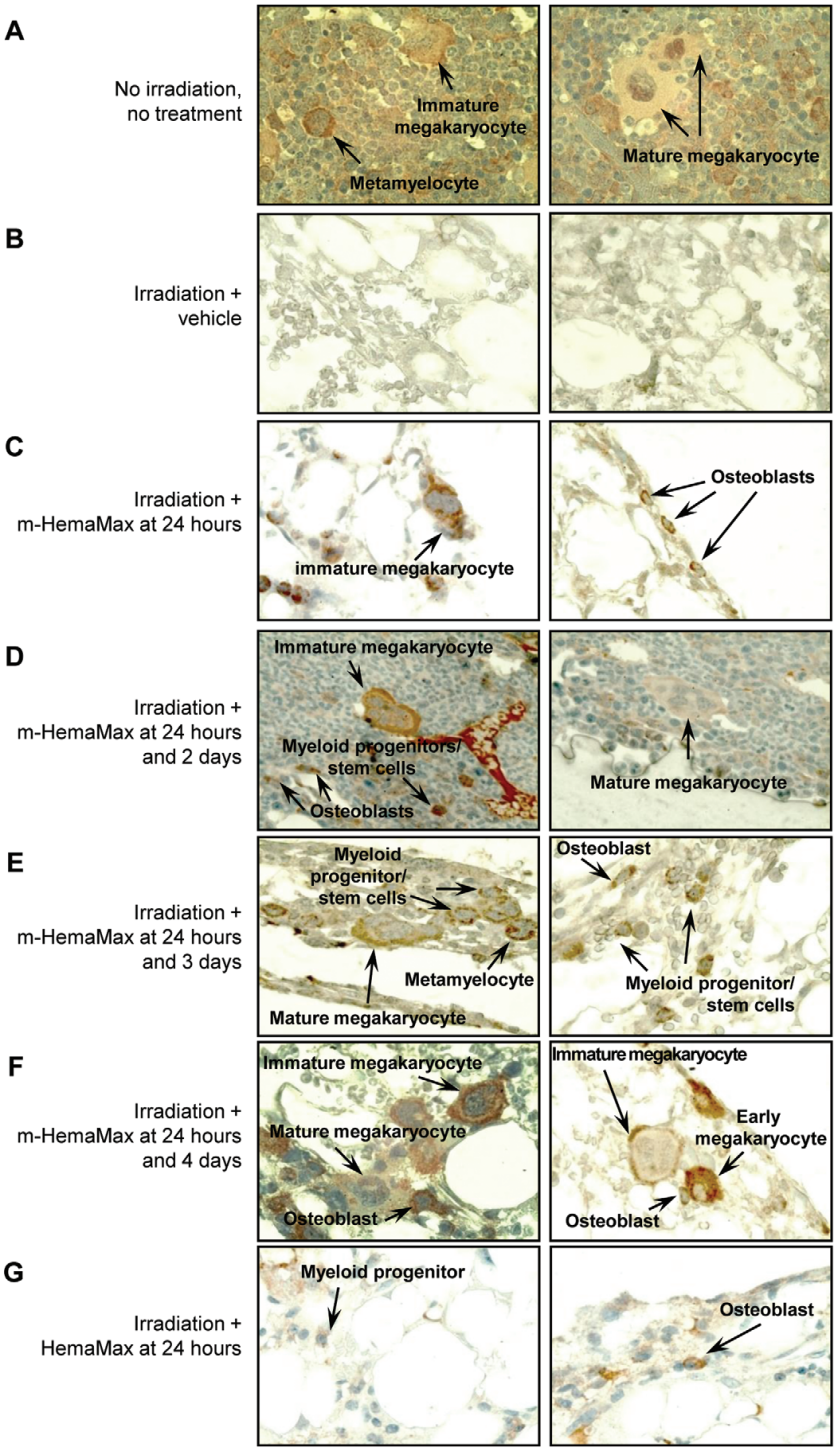
m-HemaMax promotes hematopoietic recovery in irradiated mice. Representative sections of femoral bone marrow from non-irradiated, untreated mice that were stained for IL-12Rβ2 (orange color) are shown in (a). Animals were subjected to TBI (8.0 Gy) and subsequently received vehicle (P5.6TT) or m-HemaMax (20 ng/mouse) subcutaneously at the indicated times post irradiation (b–f). An additional group of mice received HemaMax at 24 hours after TBI (g). Femoral bone marrow was immunohistochemically stained for IL-12Rβ2 (orange color) 12 days after irradiation. While bone marrow from mice treated with vehicle lacked IL-12Rβ2–expressing cells and showed no signs of hematopoietic regeneration (b), mice treated with m-HemaMax showed hematopoietic reconstitution and the presence of IL-12Rβ2–expressing megakaryocytes, myeloid progenitors, and osteoblasts (c–f). Mice treated with HemaMax showed IL-12Rβ2–expressing osteoblasts but lacked megakaryocytes (g). Magnification = 100×.

Femoral bone marrow from irradiated mice treated with vehicle or m-HemaMax ≥24 hours after TBI (LD_30/30_) were stained for IL-12Rβ2 and evaluated for histological signs of recovery from radiation-induced injury at 12 days post TBI. As a control, bone marrow from non-irradiated, untreated mice was characterized with the presence of IL-12Rβ2–expressing hematopoietic stem cells, identified by co-staining for Sca-1 (a murine stem cell marker; see below), immature megakaryocytes with lobulated nuclei surrounded by a narrow rim of cytoplasm, matured megakaryocytes with lobulated nuclei and voluminous cytoplasm, and myeloid progenitor cells in the metamyelocyte stage (Figure 5a).

Bone marrow from mice treated only with vehicle and subjected to an LD_30/30_ of TBI (8.0 Gy) was characterized with minimal signs of hematopoietic regeneration and the complete lack of IL-12Rβ2–expressing cells after 12 days following irradiation (Figure 5b). In contrast, mice treated with various dosing regimens of m-HemaMax showed varying levels of hematopoietic reconstitution, which was characterized with the presence of IL-12Rβ2–expressing myeloid progenitors, megakaryocytes, and osteoblasts (Figure 5c–f). Mice treated with HemaMax, which has been demonstrated to not cross react with the murine IL-12 receptor, showed some signs of regeneration, however, lacked megakaryocytes (Figure 5g). For mice treated with HemaMax, however, no increase in the survival was observed, as compared with the vehicle control group (data not shown).

**Figure 6:**
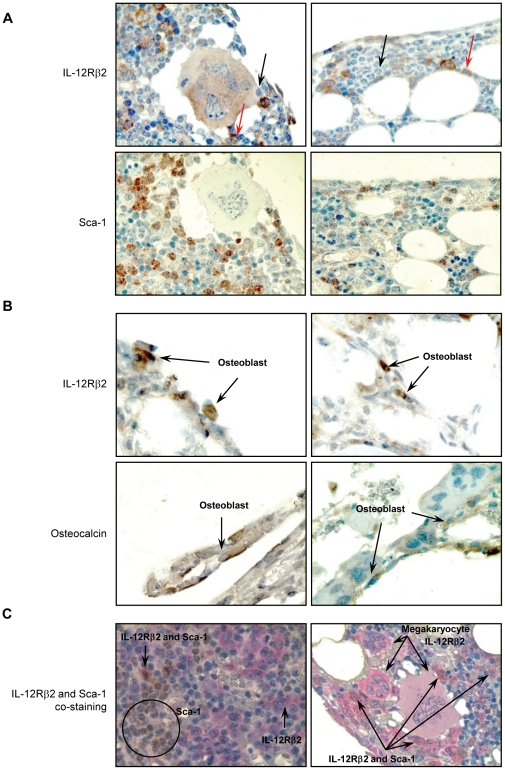
Mice bone marrow hematopoietic stem cells, osteoblasts, and megakaryocytes express IL-12Rβ2. Tissue sections obtained 30 days (a and c) and 12 days (b) after TBI (according to the protocol described in Figure 5) were stained immunohistochemically for IL-12Rβ2 (a and b, upper panels), markers of hematopoietic stem cells, Sca-1 (a, lower panel), and osteoblasts, osteocalcin (b, lower panel), or both IL-12Rβ2 and Sca-1 (c). Also both immature and mature megakaryocytes showed intense immunohistochemical staining for the presence of IL-12Rβ2 (c). Red arrows in (a) indicate hematopoietic stem cells that express IL-12Rβ2 while black arrows indicate those that do not express IL-12Rβ2. In IL-12Rβ2 and Sca-1 double staining (c) IL-12Rβ2 is stained pink while Sca-1 is stained brown. The subpopulation of stem cells co-expressing IL-12Rβ2 and Sca-1 as well as subpopulations expressing only IL-12Rβ2 or Sca-1 are indicated (c). Magnification = 100×.

In order to further evaluate as to whether morphologically identified cells were indeed hematopoietic stem cells and osteoblasts, bone marrow tissue sections were stained for the corresponding markers, respectively, Sca-1 and osteocalcin. As depicted in Figure 6 a and b, IL-12Rβ2 expression was observed on cells that were morphologically identified as hematopoietic stem cells and osteoblasts, which expressed Sca-1 and osteocalcin, respectively. Co-expression of IL-12Rβ2 and Sca-1 in bone marrow tissue sections was also evaluated by a dual staining approach. As depicted in Figure 6c, a discrete subset of hematopoietic stem cells were co-stained for the presence of both IL-12Rβ2 and Sca-1. Both immature and mature megakaryocytes expressing IL-12Rβ2 was also evident in the bone marrow tissue sections (Figure 6c). These findings suggest a direct role for IL-12 signaling pathway in hematopoietic reconstitution.

**Figure 7:**
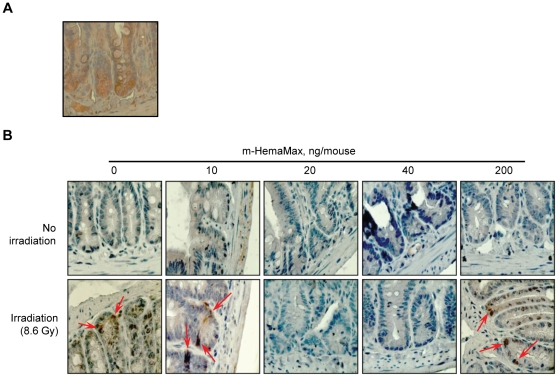
m-HemaMax at low dose suppresses radiation-induced intestinal injury in mice. The IL-12Rβ2 expression in jejunal crypts (a) and the suppression of jejunal expression of LGR5 (b), a GI stem cell injury marker, are shown. Mice received vehicle (P5.6TT) or m-HemaMax subcutaneously at the indicated doses either in the absence of irradiation or 24 hours after TBI (8.6 Gy). Three days after irradiation, jejunum tissues were removed and immunohistochemically stained for IL-12Rβ2 (a) or LGR5 (b). Representative images show LGR5 in brown as indicated with arrows. Magnification = 400.

Similar to hematopoietic stem cells and osteoblasts in femoral bone marrow, mice jejunal crypts expressed IL-12Rβ2 (Figure 7a). In the absence of irradiation, m-HemaMax administration at doses up to 200 ng/mouse did not cause injury in jejunal crypts (Figure 7b, upper panel). Exposure to TBI (8.6 Gy), however, resulted in substantial jejunal damage 3 days after irradiation, as evidenced by the widespread expression of LGR5, a GI stem cell marker shown to be expressed upon chemotherapy-induced GI injury [30]–[32]. Remarkably, administration of m-HemaMax at the low dose range of 10 ng/mouse to 40 ng/mouse dose-dependently mitigated radiation-induced jejunal damage, with no LGR5 expression evident at the optimal, efficacious dose of 20 ng/mouse (Figure 7b, lower panel). On the other hand, m-HemaMax at the high dose of 200 ng/mouse exacerbated jejunal injury (Figure 7b, lower panel). As observed with the m-HemaMax dose ranges for optimal increases in survival, these data point to a window of opportunity for mitigation of radiation injury by m-HemaMax in a very low dose range of the drug that is also effective in alleviating bone marrow damage.

### Allometric Dose Conversion From Mice to NHP

In order to achieve a similar radiomigitation effect in rhesus monkey, doses that are pharmacologically equivalent to those given to mice should be administered to rhesus monkeys. Based on the Food and Drug Administration (FDA) guidelines [28], the optimal 20 ng/mouse dose (1000 ng/Kg) and a non-optimal 80 ng/mouse (4000 ng/Kg) dose in mouse translate, respectively, to the 250 ng/Kg and 1000 ng/Kg doses in rhesus monkey. However, eliciting a pharmacologically equivalent response at species-specific equivalent doses depends on several factors including similar drug exposure and specific reactivity with the primary target site in both species. Therefore, prior to evaluating the efficacy of the radiomitigation effects of HemaMax in NHP, we first examined the pharmacological equivalency of the species-specific equivalent doses.

### HemaMax and m-HemaMax Potently Stimulated IFN-γ Secretion From Human, Rhesus Monkey, and Mouse CD14- PBMC *In Vitro*

Target reactivity to HemaMax was evaluated by comparing EC_50_ values of HemaMax and m-HemaMax for stimulating the secretion of IFN-γ from CD14- PBMC. As reported previously [33], we observed that HemaMax did not cross-react with PBMC isolated from mouse and rat (EC_50_>1000 pM). In contrast, HemaMax potently stimulated IFN-γ secretion from both human and rhesus monkey PBMC with EC_50_ values of, respectively, 2.51±0.51 pM and 1.05±0.10 pM. The EC_50_ value of m-HemaMax for stimulating IFN-γ secretion from mouse PBMC was 0.35±0.29 pM. These findings suggest that the reactivities of monkey and mouse PBMC to, respectively, HemaMax and m-HemaMax are similar in relation to IFN-γ secretion in vitro.

### Plasma PK of HemaMax in Rhesus Monkeys

**Figure 8:**
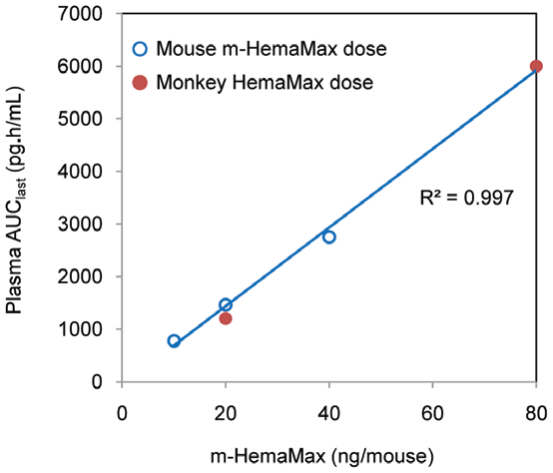
Similar exposures to m-HemaMax and HemaMax at species-specific equivalent doses in mice and rhesus monkeys. The plot of plasma AUC_last_ of m-HemaMax versus the dose administered to mice in the absence of irradiation was linear at doses from 10 ng/mouse to 40 ng/mouse. The plasma AUC_last_ of HemaMax at monkey equivalent doses of 20 ng/Kg and 80 ng/Kg was in good agreement with the extend of dose-dependent increases in m-HemaMax exposure in mice.

Plasma PK of HemaMax was examined in rhesus monkeys following a single administration of HemaMax at two doses of 250 ng/Kg and 1000 ng/Kg in the absence of irradiation. Following administration, the exposure (AUC_last_) to HemaMax increased in proportion to dose (Table 2). The AUC_last_ of HemaMax in rhesus monkey was perfectly superimposed linearly for the AUC_last_ of m-HemaMax in mice over the dose range of 10 ng/mouse to 80 ng/mouse (Figure 8), suggesting that the species-specific equivalent doses calculated from mice studies provided similar drug exposure in monkeys. The 200 ng/mouse dose was not included in this analysis as it appeared that m-HemaMax exhibits different PK characteristics at higher doses (Table 1).

**Table 2:** Plasma Pharmacokinetic Characteristics of HemaMax in Non-Irradiated Rhesus Monkeys.

HemaMax dose, ng/Kg	C_max_ (pg/mL)	AUC_last_ (pg.h/mL)	T_max_ (hours)	t_1/2_ (hours)
250	38.3±8.4	1192±382	10±3.5	20.4±12.3
1000	193.3±61.3	5708±1488	8±3.5	40.6±24.1

Animals received HemaMax subcutaneously at a dose of either 250 ng/Kg or 1000 ng/Kg in the absence of irradiation. The plasma concentrations of HemaMax were determined by ELISA.

AUC = area under the curve; C_max_ = maximum plasma concentrations; T_max_ = time to achieve the maximum plasma concentration; t_1/2_ = half life.

HemaMax at a single dose of 250 ng/Kg or 1000 ng/Kg was well tolerated and was not associated with overt signs of toxicity, except for the occurrences of transient decreases in appetite in the 1000 ng/Kg group.

### HemaMax Administration Increased Plasma Concentrations of IFN-γ, IL-15, IL-18, Neopterin, and EPO In Non-Irradiated Rhesus Monkeys

**Figure 9:**
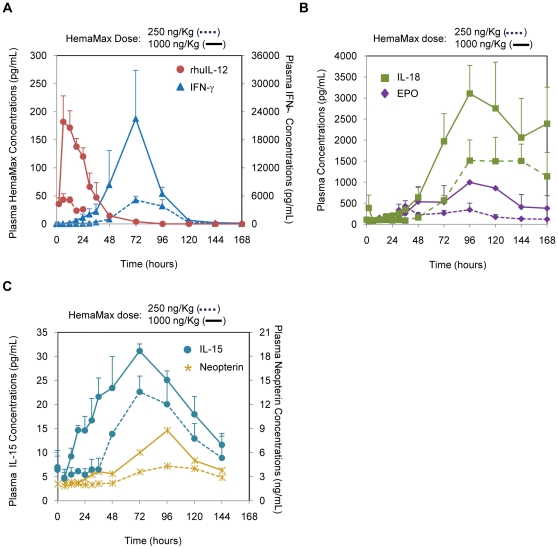
HemaMax administration increased plasma IFN-γ, IL-18, EPO, IL-15, and neopterin concentrations in non-irradiated rhesus monkeys. (a) Temporal kinetics of IFN-γ relative to that of HemaMax. (b) Temporal kinetics of IL-18 and EPO. (c) Temporal kinetics of IL-15 and neopterin. Animals received HemaMax subcutaneously at a dose of either 250 ng/Kg or 1000 ng/Kg in the absence of irradiation. The plasma concentrations of HemaMax, IFN-γ, IL-18, EPO, IL-15, and neopterin were determined by ELISA in blood samples withdrawn at the indicated times. n = 3 per timepoint in each group, except for neopterin, which was n = 1.

In monkeys, subcutaneous administration of HemaMax appeared in plasma shortly after administration and was not detectable after 72 hours (Figure 9a). Moreover, as observed in mice with m-HemaMax, HemaMax was observed to increase plasma IFN-γ concentration in proportion to dose (Figure 9a). Temporal kinetics of IFN-γ response in rhesus monkey was, however, different from mouse in that the IFN-γ response was delayed for a longer period of time and was much higher in magnitude (Figure 9a). Neither HemaMax, nor IFN-γ, was detected in plasma of monkeys that did not receive HemaMax.

Of other potential biomarkers, the exposure (AUC_last_) to IL-18 and EPO was increased by 2.4-fold and 5.1-fold, respectively, as the HemaMax dose was increased from 250 ng/Kg to 1000 ng/Kg (Figure 9b). HemaMax also increased plasma IL-15 and neopterin concentrations, peaking at 72 hours and 96 hours, respectively, post HemaMax administration (Figure 9c). In contrast to previous reports in humans [34]–[37], the plasma concentrations of rhesus monkey TNF-α and IL-10 were not changed. Numerous other potential biomarkers were screened, but were found to be below the limit of quantification.

### NHP and Human Bone Marrow and Small Intestine Express IL-12Rβ2

**Figure 10:**
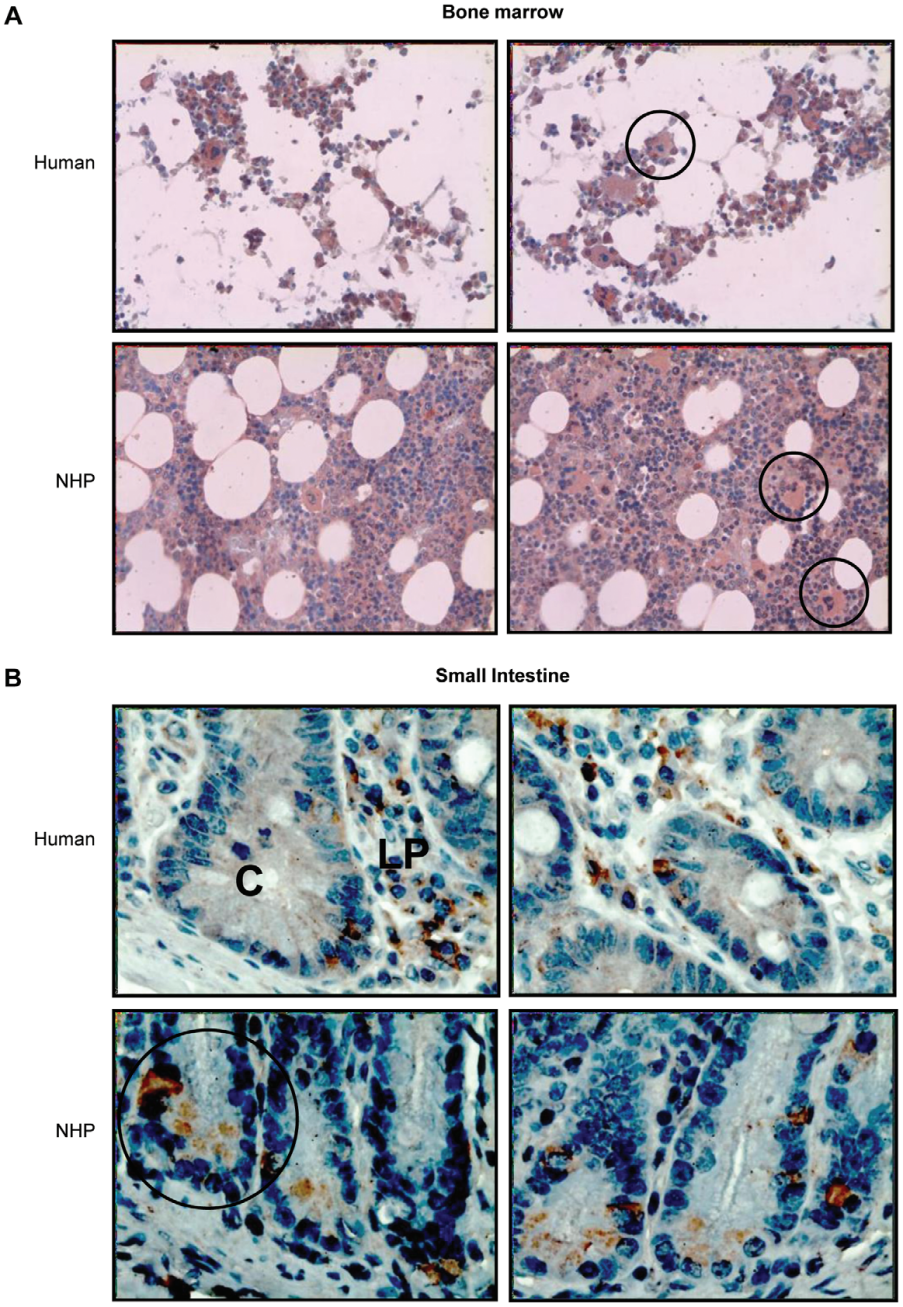
NHP and human bone marrow and small intestine express IL-12Rβ2. Tissues from NHP and human femoral bone marrow (a) and jejunum/ileum (b) were immunohistochemically stained for IL-12Rβ2. (a) Progenitor cells and megakaryocytes expressing IL-12Rβ2 are shown. Adipocytes did not express IL-12Rβ2. (b) Intestinal crypts expressing IL-12Rβ2 are shown. Lymphoid cells in the lamina propria and submucosal regions also expressed IL-12Rβ2. C = crypt; LP = lamina propria. Magnification was 40× in (a) and 100× in (b).

The expression of IL-12Rβ2 in non-irradiated NHP (rhesus monkeys) and human femoral bone marrow and jejunum/ileum was evaluated by immunohistochemistry. As depicted in Figure 10a, NHP, as well as human, progenitor cells and megakaryocytes expressed IL-12Rβ2. The expression of IL-12Rβ2 was also found on osteoblasts/osteoclasts from the bone marrow. However, it could not be determined as to whether these cells were osteoblasts and/or osteoclasts because the donated tissues were smears and did not include periosteum or other bone tissues. Bone marrow adipocytes were not stained positive for IL-12Rβ2.

In the small intestine, IL-12Rβ2 was most commonly expressed in crypts (Figure 10b). It is not known if IL-12Rβ2-expression in the intestinal crypt is localized to Paneth cells, multipotent stem cells, or both. IL-12Rβ2 expression was also noted in lymphoid cells populating the lamina propria and submucosal regions (Figure 10b). Mucin secreting goblet cells did not express IL-12Rβ2. Both crypt and lamina propria IL-12Rβ2-expressing cells could represent multifunctional mesenchymal-origin myofibroblasts that can serve as crypt shape-forming cells that also occupy both a stem cell niche and act as non-professional antigen presenting cells to immunomodulatory cells in the lamina propria. Further studies will establish the cellular and functional identity of IL-12Rβ2-expressing cells in intestinal crypts and their supportive role in intestinal regeneration after radiation exposure.

### HemaMax Administration Increased Survival in Irradiated, Unsupported Rhesus Monkeys

**Figure 11:**
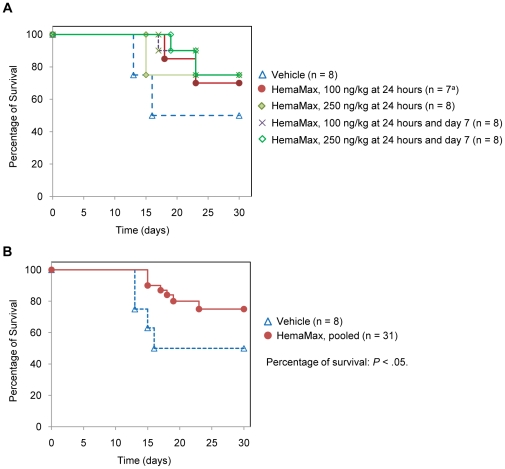
HemaMax initiated at least 24 hours after irradiation increased percentage of survival of unsupported monkeys. Individual dosing groups (a) and the pooled HemaMax dosing group (b) are shown. Animals were subjected to an LD_50/30_ of TBI at day 0 and subsequently received either vehicle (P5.6TT) or HemaMax subcutaneously at the indicated dosing regimens. Supportive care was prohibited during the study. Animals were monitored for survival up to 30 days. ^a^ One animal was excluded from the study due to a broken tooth.

In a pilot study of 40 animals, the percent survival of rhesus monkeys exposed to an LD_50/30_ of TBI ( 6.7 Gy) was determined following treatment with 100 ng/Kg or 250 ng/Kg of HemaMax administered at 24 hours or at 24 hours and 7 days post TBI. This study was conducted in the absence of any supportive care, including antibiotics. The doses of HemaMax were chosen based on PK/PD studies in rhesus monkeys and were equivalent to m-HemaMax doses of 8 ng/mouse and 20 ng/mouse, respectively. As is depicted in Figure 11a, HemaMax at both doses, following either single or two administrations, migitated death due to irradiation to the same extent. Overall percentages of survival were 71% in the 100 ng/Kg single dose group (n = 7) and 75% in all other groups receiving HemaMax (n = 8) compared to 50% in the vehicle group. Between-group differences in percentage of survival were not statistically significant, most likely because of the small number of animals in each group (n = 8), but also because both HemaMax doses were likely within the efficacious dose range. However, analysis of the percent survival regardless of the HemaMax dosing regimen indicated that when pooled together, monkeys treated with HemaMax had significantly higher percent survival than those receiving vehicle (75% vs 50%, respectively; *P* = .05) (Figure 11b).

### Changes in Blood Cell Counts of Irradiated, Unsupported Rhesus Monkeys Following HemaMax Administration

**Figure 12:**
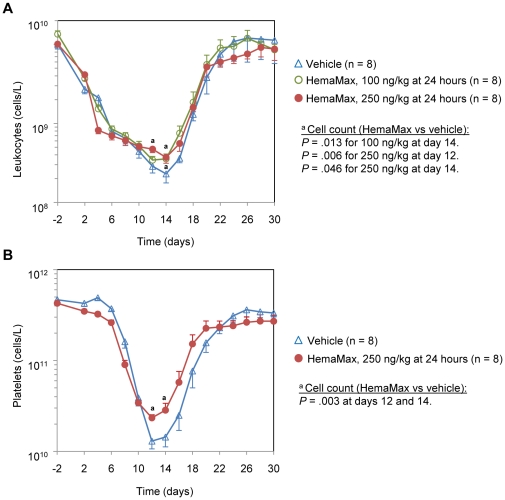
HemaMax administration decreased leukopenia (a) and thrombocytopenia (b) at nadir in irradiated, unsupported rhesus monkeys. Animals were subjected to an LD_50/30_ of TBI at day 0. Animals received subcutaneously either vehicle (P5.6TT) or HemaMax at a dose of 100 ng/Kg or 250 ng/Kg at 24 hours post TBI. Blood samples were withdrawn at the indicated times, and leukocytes and platelets were counted by an automated hematology analyzer.

Three analyses were conducted to assess differences in blood cell counts during the study period. In the first analysis, where blood cell counts were analyzed from day 1 up to day 30, animals treated with HemaMax had significantly higher numbers of leukocytes and thrombocytes at days 12 and 14, around the nadir, for the 100 ng/Kg and 250 ng/Kg doses, as compared to animals treated with vehicle (Figure 12).

In a second analysis, where blood cell counts were analyzed from day 1 up to day 14, the day before any animals died, animals treated with HemaMax had higher platelets counts compared to animals treated with vehicle (*P* = .079 for the 250 ng/Kg group and *P* = .02 for the 100 ng/Kg twice dosing group) during nadir (days 12 to 14). Additionally, in comparison to the vehicle group, animals treated with HemaMax had significantly higher counts of leukocytes (*P*<.01 for the 250 ng/Kg group and *P*<.04 for the 100 ng/Kg twice dosing group) and reticulocytes (*P*<.04 for the 250 ng/Kg group and *P*<.001 for the 100 ng/Kg group) during nadir (days 12 to 14). The same trend was apparent for neutrophil, basophil, and lymphocyte counts, but they did not reach acceptable levels of statistical significance.

In a third analysis the number of animals that reached clinically low platelet counts during the study was assessed. This analysis revealed a remarkable difference between the vehicle and HemaMax groups in the number of platelet counts dropping below a threshold level of 20,000 platelets/µL, a level generally necessitating platelet transfusion. In the HemaMax 250 ng/Kg group, only 4 out of 16 (25%) platelet counts at the nadir (day 12 to day14) dropped below the transfusion threshold of less than 20,000 platelets/µL whereas 12 out of 15 (80%) platelet counts for the vehicle animals were below the threshold level during the same period of time (*P* = .007).

Taken altogether, these findings indicate that HemaMax increases leukocytes, platelet, and reticulocyte counts just prior to the days on which animals begin to die from radiation toxicity (day 13, Figure 11a). Interestingly, vehicle-treated animals that survived up to day 30 also had quick recovery of blood cell counts, which were statistically indistinguishable from those in the HemaMax groups. These findings suggest that mortality likely occurs in animals that do not show a strong blood cell recovery around the nadir day(s). The validity of this hypothesis was evaluated by comparing blood cell counts of animals stratified by the mortality status, *i.e.*, those surviving up to day 30 versus animals dying after day 12. In this analysis, the blood cell counts on the day before death was taken for animals that died after day 12. The comparison day for the surviving animals in each group was the average day on which the decedents in a particular group died (days 14 to 18). This analysis demonstrated that, regardless of the particular treatment group, animals surviving up to day 30 had significantly higher counts of platelets, neutrophils, leukocytes, reticulocytes, and lymphocytes than those that died after day 12 (*P*<.001 to *P*<.05). When compared by treatment group, animals treated with 100 ng/Kg HemaMax had significantly higher counts of neutrophils, leukocytes, and lymphocytes than did those treated with vehicle in both survivors and decedent groups (*P*<.001 for all three cell types). In addition, animals treated with 100 ng/Kg HemaMax had a numerically higher platelet and reticulocyte counts. These findings suggest that HemaMax-induced increase in blood cell counts around nadir may play a key role in promoting survival following radiation exposure.

### Clinical and Physical Characteristics of Irradiated, Unsupported Rhesus Monkeys Following HemaMax Administration

**Figure 13:**
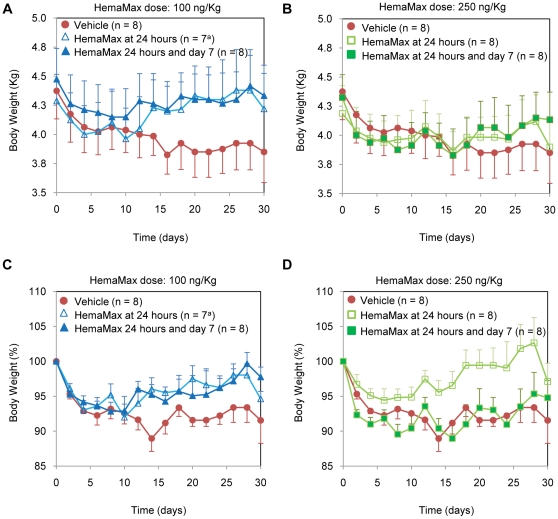
Irradiated rhesus monkeys receiving HemaMax had less body weights loss than animals receiving vehicle. Body weights in Kg (a and b) and in percentage (c and d) are shown for the 100 ng/Kg and 250 ng/Kg dose groups. Monkeys were subjected to an LD_50/30_ of TBI at day 0 and subsequently received either vehicle (P5.6TT) or HemaMax subcutaneously at the indicated dosing regimens. Supportive care was prohibited during the study. Body weights were recorded every other day for up to day 30.

Animals receiving HemaMax at the dose of 100 ng/Kg (once or twice) had consistently higher mean body weights than did those in the vehicle group from days 14 to day 30 (Figure 13a). Animals treated with HemaMax at the dose of 100 ng/Kg (once or twice) or 250 ng/Kg (once) had less weight loss than did animals treated with vehicle from days 14 to 30 (Figure 13 c and d). Although the between group differences in body weight or weight loss were not statistically significant, when the analysis of body weight loss was limited to day 12—the approximate day for blood cell nadir and the day after which animals began to die (Figure 11a)— the pooled HemaMax-treated animals had significantly less body weight loss than those treated with vehicle (95.3±0.8% versus 91.6±1.5%, respectively; *P* = .04). Logistic regression demonstrated that weight loss after day 12 was a strong predictor of survival (*P*<.001). Other clinical signs (appetite, physical activity, diarrhea, and feces color) were not significantly different from the vehicle group, although appetite and physical activity improved in HemaMax treated animals, and the incidence of diarrhea and black or red feces declined in the 250 ng/Kg twice dosing regimen group. However, above-mentioned clinical signs did predict mortality after day 12 by logistic regression (*P* = .002 for decreased appetite, *P*<.001 for decreased physical activity, *P* = .04 for incidence of diarrhea, and *P* = .008 for incidence of red or black feces). Clinical signs of severe deterioration and stress, including chronic anorexia, sunken eyes, dehydration, hunched and/or crouching posture and weakness, started approximately at day 14 with no remarkable between-group differences in the incidence or onset. All adverse clinical signs were consistent with acute radiation syndrome following exposure to radiation.

Gross pathology along with organ and hemoculture bacteriology evaluation was conducted for all animals, which died or were euthanized before the end of study. There were no HemaMax–related macroscopic lesions. The incidence of hemorrhage was 12.5% (1/8 animals) in the pooled animals treated with 100 ng/Kg or 250 ng/Kg of HemaMax compared to 50% (2/4) in the vehicle animals. In the vehicle group, all of the decedent animals (4/8 animals) were found dead while only 1 animal in the HemaMax groups was found dead and 8 animals were humanely euthanized before the end of study. A diagnosis of septicemia was confirmed by isolation of the same bacterial strain in at least 2 organs of all 13 animals.

In the vehicle group, 75% (3/4) found dead animals presented a combination of bacteria most likely from the intestinal and cutaneous flora and 25% (1/4) presented organ infections with only bacteria most likely from the cutaneous bacterial flora. In the various HemaMax-treated groups, 8 out of 9 animals (89%) presented a combination of bacteria from the intestinal and cutaneous flora, including 2 which also presented organ infections with bacteria most likely from the environment. The other animal (1/9) presented organ infections with only bacteria most likely from the cutaneous flora. These results suggest that opportunistic infections were present in all animals that died preterminally in this animal model of acute radiation syndrome.

## Discussion

Radiation toxicity caused by TBI dose-dependently implicates immune, hematopoietic and the GI tissues, as these are the most radiosensitive targets in the body. Lymphocytes are the most sensitive cells to radiation toxicity and, at irradiation doses of >2 Gy, are the first to be depleted from circulation. The lymphocyte loss is followed by a decline in granulocytes and then platelet levels over a period of days. Acute-onset anemia may occur secondary to hemorrhage [1], [2]. At doses of >4 Gy, radiation adversely affects GI epithelium/endothelium, and the resulting clinical manifestation is due to a combination of the hematopoietic and GI toxicities, presenting with nausea, vomiting, diarrhea, headache, fatigue, fever, and abdominal pain [1], [2]. Death originating from immune and hematopoietic toxicity occurs because of infection due to impaired immunity and/or hemorrhage due to thrombocytopenia, while death originating from GI toxicity is often because of multisystem organ failure, overwhelming sepsis, and complications of bleeding [2]. In the event of a radiological attack, radiation mitigators with multi-tissue effects capable of alleviating immune, hematopoietic and GI toxicities when administered after radiation exposure, are needed to save lives.

Our findings demonstrate that HemaMax (this term is used throughout this discussion to collectively include its prototype m-HemaMax as well) mitigated death due to radiation-induced toxicity in both mice and monkeys following administration of a single, low dose. Importantly, in both mice and monkeys, HemaMax increased survival when administered at protracted timepoints post radiation exposure, such as 24 hours or longer, in the absence of supportive care, including oral or topical antibiotics. In irradiated mice and monkeys, HemaMax promoted survival at various levels by stimulating the immune system in the peripheral blood and extravascular spaces, promoting hematopoietic regeneration in bone marrow, decreasing tissue injury in the small intestine, and triggering a generalized anti-apoptotic and anti-inflammatory effect throughout the body.

The optimal murine dose that provided these radiomitigation effects is approximately 20 ng/mouse. This dose is ostensibly lower than our previous reports for HemaMax efficacy in radioprotection and as a hematological adjuvant in cancer therapy [13], [14]. However, the discrepancy is likely due to the use of formulated protein in the present studies. Moreover, protracted administration of HemaMax at 24 hours post irradiation appears to act via a somewhat different mechanism as compared with our previous studies where HemaMax was administered either before, or shortly after, radiation exposure [13], [14]. Evidence for this statement comes from a comparison of the bone marrow recovery in the current murine radiomitigation studies, as compared to previous studies in mice [14], [38]. In the current studies, bone marrow recovery appeared to be much slower, likely due to the timing of HemaMax administration (24 hours before TBI in previous studies versus 24 hours after TBI in this study).

Further, in the current radiomitigation studies in mice, when compared to irradiated control, HemaMax markedly decreased the radiation induced expression of LRG5, a stem cell marker which also serves as a marker of GI injury, when administered at 24 hours post radiation exposure. HemaMax, at doses from 10 ng/mouse to 40 ng/mouse administered 24 hours post TBI, reduced radiation-induced LGR5 expression. In contrast, with 200 ng/mouse HemaMax administration 24 hours after TBI, appeared to exacerbate radiation-induced GI injury as evidenced by an increase in LGR5 expression.. This finding may be consistent with earlier reports that high doses of IL-12 exacerbated radiation injury to the GI tract [14], [38]. Data obtained in both mice (data not shown) and rhesus monkeys show significant increases in body weights for m-HemaMax and HemaMax-treated animals, respectively,after irradiation (Figure 13), thereby providing further support for the protective GI effect of of HemaMax treatment.

Our findings that HemaMax can reduce radiation toxicity and increase survival in mice was confirmed in monkeys. HemaMax administered to rhesus monkeys at 24 hours post radiation significantly increased survival (*P* = .05, pooled treated groups vs. vehicle control). HemaMax-treated monkeys had significantly higher numbers of platelets, leukocytes, and reticulocytes at the nadir, had lower incidence of hemorrhage, and had higher body weights from day 12 to day 30.

To provide perspective about the importance of body weight changes, it is worth mentioning that weight loss after day 12 is a strong predictor of survival (*P*<.001). The lack of statistically significant between-group differences in the incidence of cause of death from hemorrhage and decreases in body weight is likely because of the small number of animals in each group. On the other hand, if the change in body weight analysis was limited to the blood cell nadir (day 12), just before animals began dying, the pooled group analysis (all HemaMax-treated groups versus the vehicle group) resulted in a statistically significant difference (*P* = .04), while the individual HemaMax-treated groups were marginally different from the vehicle group (*P* = .078). Moreover, thrombocytopenia was less severe in animals treated with HemaMax than in those treated with vehicle. Further, a remarkable difference was observed between the vehicle and HemaMax-treated groups in platelet counts dropping below the threshold level of 20,000 platelets/µL, a level that generally necessitates platelet transfusion. In the HemaMax-treated 250 ng/Kg group, only 4 out of 16 (25%) platelet counts at the nadir (day 12 to day14) dropped below the transfusion threshold of less than 20,000 platelets/µL whereas 12 out of 15 (80%) platelet counts for the vehicle animals were below the threshold during the same period of time (*P* = .007). Thus, in spite of small number of animals in the monkey study, statistically significant differences were observed in several key survival-related parameters. These findings are significant as, in our study, HemaMax was administered after TBI, the earliest at 24 hours post irradiation—a window of time considered minimally necessary for mobilization of medical personnel and resources to the affected area. This is the first study that demonstrates the potential of HemaMax as a life-saving intervention in the event of a radiological disaster.

Our findings reported here provide evidence that when administered as single, low doses after TBI, HemaMax mitigates radiation-induced toxicity in at least three major systems affected by radiation: the immune system, the bone marrow compartment, and the GI tract. HemaMax protection of the GI tract,however, remains to be confirmed in GI focused experiments implemented at higher radiation levels. An additional event related to the HemaMax mitigation of radiation toxicity is the stimulation of anti-apoptotic/anti-inflammatory effects via release of EPO, a known general protector of tissue against cytotoxic damage via anti-apoptotic/anti-inflammatory mechanisms [74].

Several interdependent networks may underlie the radiomitigation effect of HemaMax. It is known that IL-12 is a central regulator of cell-mediated immune responses and modulates the synthesis and secretion of several immune mediators [39], [40]. In cancer patients, intraperitoneal/intravenous/subcutaneous administration of IL-12 increased peritoneal/serum levels of IFN-γ, TNF-α, IL-10, IL-8, VEGF, IP-10, and neopterin [34]–[37]. In our study, HemaMax administration dose-dependently increased plasma IFN-γ levels in both mice and monkeys. IFN-γ orchestrates many distinct cellular programs through transcriptional control over large numbers of genes, resulting in heightened immune surveillance and immune system efficiency against infection [41]. In addition to IFN-γ, HemaMax increased plasma levels of EPO in mice (IL-15, IL-18 and neopterin were not tested) and IL-15, IL-18, neopterin, and EPO in monkeys. The lack of dose proportional response with IL-15, IL-18 and neopterin is likely due to saturation of target sites related to release of these mediators. However, the lack of a dose proportional response for EPO appears to be more related to an interplay between IFN-γ and EPO, as suggested by our data and the reports of others [42], [43]. In a previous report, administration of EPO has been shown to suppress the inflammatory response related to IFN-γ in an animal model of multiple sclerosis [29].

IL-15 and IL-18, alone and/or in combination, play important roles in the development, homeostasis, and functions of CD4+ T cells, CD8+ T cells, natural killer (NK) cells, and NK T cells [44]–[46]. In synergism with IL-12, IL-18 stimulates the production of IFN-γ in T helper 1 cells [47]. Neopterin, an auto-oxidation product of 7,8-dihydroneopterin, reflects IFN-γ activity and, as a corollary, is considered as an indicator of systemic immune activation [48], [49].

The current study is the first to demonstrate that HemaMax stimulates EPO production in mice and NHP. This finding suggests that EPO may play a central role in mediating the radiomitigation activity of HemaMax. The role of EPO in biological functions other than erythropoiesis has only recently begun to unravel, primarily after finding EPO receptors on cells other than erythroid progenitors, such as polymorphonuclear leukocytes, megakaryocytes, and endothelial, myocardial, and neuronal cells [50]–[55]. Accumulating evidence indicates that EPO has immunomodulatory, neuroprotective, and cardioprotective activities. EPO enhances cell viability, modulates surface antigen expression, and increases IL-12 secretion in dendritic cells–the most potent antigen presenting cells–suggesting that immunomodulatory functions of EPO may partly be mediated through dendritic cells, which in turn induce specific T cell responses [56], [57]. Cytoprotective effects of EPO have at least in part, been linked to its antioxidant, anti-inflammatory and antiapoptotic activities. In various models of cytotoxicity induced by toxicants, ischemia, hypoxia, or oxidative stress, EPO increased cellular antioxidant capacity and/or decreased oxidant injury in kidney, neurons, and retinal pigment epithelial cells [58]–[62] while it reduced apoptosis in neurons, vascular smooth muscle cells, cardiomyocytes, and endothelial cells [63]–[66].

**Figure 14:**
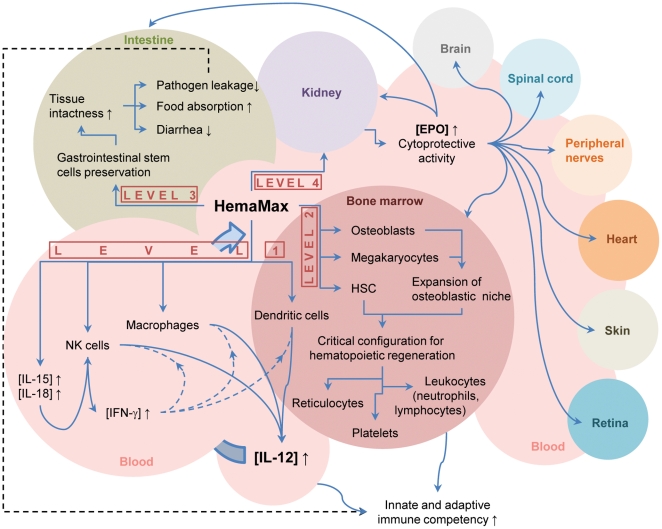
A multilevel model of HemaMax mechanism of action in increasing survival following exposure to radiation. Current evidence suggests that HemaMax triggers responses at, at least, 4 levels in the body. At the Level 1 response, HemaMax promotes proliferation and activation of extant, radiosensitive immune cells, namely NK cells, macrophages, and dendritic cells. HemaMax-induced plasma elevations of IL-15 and IL-18 also facilitate maturation of NK cells, leading to the release of IFN-γ, which in turn, positively affects the production of endogenous IL-12 from macrophages and dendritic cells, and perhaps NK cells. These events enhance the innate immune competency early on following HemaMax administration. At the Level 2 response, HemaMax promotes proliferation and differentiation of the surviving hematopoietic stem cells, osteoblasts, and megakaryocytes into a specific cellular configuration that ensues optimal hematopoiesis. HemaMax-induced secretion of EPO from CD34+, IL-12Rβ2–positive bone marrow cells may also suppress local over-production of IFN-γ in the bone marrow and, thus, provide a milieu that promotes expansion of hematopoietic cells. Hematopoietic regeneration in the bone marrow enhances both innate and adaptive immune competency. At the Level 3 response, HemaMax preserves GI stem cells, leading to a reduction in pathogen leakage, an increase in food consumption, and a decrease in diarrhea. At the Level 4 response, HemaMax likely directly increases renal release of EPO, a cytoprotective factor, which enhances cellular viability in a diverse set of organs/tissues. Continued production of endogenous IL-12 primarily from dendritic cells activated by pathogens and/or EPO serves as a positive feedback loop and plays a key role in sustaining the initial response to exogenous HemaMax, perhaps for weeks after radiation. ↑ = increase; ↓ = decrease; HSC = Hematopoietic stem cells; NK cells = natural killer cells.

The underlying mechanisms of radiomitigation conferred by exogenous HemaMax is now beginning to be revealed by the findings of our current and previous studies, suggesting a multilevel response orchestrated by exogenous delivery of HemaMax (Figure 14). Current evidence suggests that HemaMax triggers responses at, at least, 4 levels by directly activating IL-12 receptors (a) on immune cells in peripheral blood and bone marrow (Level 1), (b) on hematopoietic stem cells and other key cells of the bone marrow niche, such as osteoblasts (Level 2), (c) on GI stem cells (Level 3), and likely (d) on kidney cells (Level 4), whereby EPO, a cytoprotective factor, is released following radiation exposure (Figure 14).

The most immediate response is the HemaMax-induced Level 1 Response, which involves key radioresistant cells of the immune system. At the very early stages following radiation exposure, most immune cells undergo apoptosis with a rank order according to their radiosensitivity (B cells>T regulatory cells>T helper cells>T cytotoxic cells>T memory cells>NK cells) [4]. The immune cells that are likely to remain functional at 24 hours or longer post irradiation are those that are the least radiosensitive, namely NK cells and differentiated cells, such as macrophages and dendritic cells. Thus, HemaMax administered after radiation can initiate the Level 1 response by promoting the proliferation and activation of the surviving NK cells, macrophages, and dendritic cells [4], [15]. The tridirectional cross-talk between NK cells, macrophages and dendritic cells further promotes their maturation and expansion via cytokines identified as biomarkers of the restoration of innate immunity, namely IFN-γ, IL-15, IL-18 and neopterin [49], [67]–[69]. This tridirectional cross-talk further leads to the production of endogenous IL-12 secreted from dendritic cells (Figure 14). As a consequence, early immune competence is established via innate immunity mechanisms following TBI. Continuous production of endogenous IL-12 from pathogen-activated dendritic cells also serves as a positive feedback loop and plays a key role in sustaining the initial response to exogenous HemaMax, perhaps for weeks after radiation exposure (Figure 14). Evidence for the continued production of endogenous IL-12 following exogenous administration of HemaMax is the presence of IL-12Rβ2 on hematopoietic cells 12 days after TBI only in mice that were treated with HemaMax.

HemaMax initiates the Level 2 Response through interaction with the primary bone marrow cells involved in hematopoiesis. In the bone marrow, residual hematopoietic stem cells, osteoblasts, and megakaryocytes are likely the cell types that remain extant and functional 24 hours following exposure to lethal doses of radiation [70]. The presence of IL-12Rβ2–expressing stem/progenitor cells, megakaryocytes, and/or osteoblasts in bone marrow from mice, NHP, and humans indicates that these cells are direct targets of HemaMax. Through its receptors, HemaMax initiates the Level 2 response by promoting proliferation and differentiation of the surviving stem cells following radiation exposure, leading to hematopoietic regeneration (Figure 14). Activation of osteoblasts appears to be crucial for the survival, expansion, and homing of hematopoietic stem cells and megakaryocytes [70]–[75]. It has been shown that exposure to lethal doses of radiation leads to a specific expansion of osteoblastic niche, whereby the surviving pool of radioresistant osteoprogenitors proliferates close to the endosteal bone areas [76]. The relatively long-lived, surviving megakaryocytes were also observed close to the endosteal surface of trabecular bone rather than in their normal parasinusoidal site. Megakaryocytes release factors that stimulated the expansion of osteoblastic niche [76]. Consistent with these findings, immunohistochemical examinations in our study revealed a similar cellular configuration in mice bone marrow, showing cellular islands consisting of osteoblastic niche, megakaryocytes, and hematopoietic stem cells close to the bone. In CD34+, IL-12Rβ2–positive bone marrow cells, HemaMax increases EPO secretion while, in contrast to its traditional action in mature lymphocytes, it decreases IFN-γ secretion [unpublished data from our lab], providing a milieu that promotes expansion of hematopoietic stem cells, eventually leading to regeneration of mature blood cells including platelets and leukocytes (Figure 14). EPO also contributes to the development of such optimal milieu by suppressing the over-production of inflammatory cytokines such as IFN-γ, IL-6, IL-2, and TNF-α from T cells [29]. Inhibition of IFN-γ production by EPO is in agreement with our findings showing that plasma IFN-γ levels were suppressed in irradiated mice at a HemaMax dose (20 ng/mouse) that substantially increased plasma EPO levels. Furthermore, the increased plasma EPO concentrations may, at least in part, explain the lack of increases in monkey plasma levels of proinflammatory cytokines such as IL-2, IL-6, and TNF-α following HemaMax administration.

HemaMax initiates the Level 3 Response by preserving GI stem cells, which regenerate intestinal crypt cells and ensure intestinal integrity (Figure 14). HemaMax-induces intestinal cell-cell border integrity, which reduces pathogen leakage, increases food absorption, and decreases diarrhea. The reduction of “leaky gut syndrome” provides further immune-related benefit by decreasing pathogen entry into peripheral blood circulation (Figure 14). HemaMax-induced GI recovery thus provides a greater chance of survival following lethal radiation exposure.

HemaMax initiates the Level 4 Response by increasing plasma levels of EPO, likely by enhancing EPO release from the kidneys following direct activation of its renal receptors. Given its antioxidant, anti-inflammatory, and antiapoptotic activities, EPO acts as a general cytoprotective factor in the body, enhancing cellular viability in a diverse set of organs/tissues including the brain, peripheral nerves, heart, kidney, skin, and intestine [77]. EPO may also preserve key cells involved in Level 1 and 2 survival advantages of HemaMax, namely niche bone marrow cells, as well as mature and immature dendritic cells, macrophages, and NK cells against radiation toxicity. Matured dendritic cells may also release IL-12 in response to EPO [56], [57] and/or IFN-γ [78], providing a positive feedback loop that amplifies the events originally initiated by exogenous administration of HemaMax.

Finally, continuous generation of endogenous IL-12 induced by a single dose of exogenous HemaMax in irradiated, immunocompromised hosts is another key survival advantage. Continuous endogenous production of IL-12 is primarily a result of the Level 1 HemaMax-induced response. In addition, bacterial and pathogenic products gaining access to the circulation following radiation injury can activate dendritic cells to promote innate and adaptive responses, and further lead to a release of endogenous IL-12. Thus, we hypothesize that HemaMax promotes proliferation of surviving immune cells, cells of the bone marrow niche, namely osteoblasts and megakaryocytes, hematopoietic stem cells, and provides protection against radiation injury to key intestinal stem cells through various feedback loops. These feedback loops promote the generation of soluble factors such as endogenous IL-12, IFN-γ, and EPO, allowing regeneration of hematopoietic system and recovery of immune and GI functions (Figure 14).

The studies reported here culminate with the important finding from several proof of concept studies, where we demonstrate for the first time that HemaMax mitigates radiation-induced injury in NHP, an animal model that is closely related to human. Importantly, for the FDA Animal Rule path to approval, allometric dose conversion from mice to rhesus monkey allowed identification of comparable doses that provided similar HemaMax exposure in monkeys. Despite similar PK characteristics, the IFN-γ response to HemaMax appeared to be stronger in monkeys compared to mice. The fact that the percentage of survival of rhesus monkeys was similar after receiving either a single dose or two doses of HemaMax at either 100 ng/Kg or 250 ng/Kg suggest that HemaMax is likely to be effective at even lower doses. Importantly, the HemaMax doses used in the NHP studies correspond to human doses of about 30 ng/Kg and 80 ng/Kg, respectively. In cancer patients, IL-12 has been administered intravenously, intraperitoneally, or subcutaneously at a dose range of 3 ng/Kg to 600 ng/Kg as a monotherapy or part of a combination therapy for the treatment of various carcinomas [34]–[36], [79], [80]. Intravenously, IL-12 has been associated with a high rate of toxicity [79]. Intraperitoneally, IL-12 reaches dose limiting toxicity (elevated transaminase) at 600 ng/Kg and is most frequently associated with fever, fatigue, abdominal pain, and nausea [80]. Subcutaneously, IL-12 is generally well tolerated when it is administered twice weekly at a range of 300 ng/Kg to 500 ng/Kg for up to 3 years [36]. In our studies, HemaMax was also well tolerated in monkeys after a single dose or up to seven doses of 1000 ng/Kg (data not shown) with no overt sign of toxicity.

Our safety studies in monkeys, coupled with the very low effective dose in both mice and monkeys, indicate that the requisite HemaMax dose for radiomitigation will be substantially lower than the IL-12 doses previously used in cancer patients, thus suggesting a more favorable safety profile for HemaMax in radiation victims Given the expected safety profile for HemaMax, it is envisioned that the drug could be disseminated to all individuals in the vicinity of a radiological event, even in the absence of any knowledge of the actual level of radiation exposure. Currently, a First-in-Human (FIH) study in healthy volunteers is ongoing.

Although the roles of IL-12 in immunity and cancer were the subject of intensive study in several clinical trials, IL-12 was never approved as a drug for any indication. The reason for the lack of advancement of IL-12 to approval was its modest clinical activity and significant toxicity due to high and repeated dosing regimens [15], [81]. As shown in this study, potent radiomitigation effects in mice and NHP can be achieved using very low, nanogram per kilogram doses of HemaMax given merely once. The single, very low dose of HemaMax required for its radiomitigation effects underscores both its potency and its expected safety in humans.

In an era of the increased risk for radiological terrorism or accident, medical contingency plans and preparedness are crucial to saving lives. The major components of such plans are the detection of radiation exposure, rapid determination of the absorbed radiation doses, and availability of the validated radiation mitigators [82]. Doubtlessly, effective radiomitigators are the key to the preparedness success. Our findings indicate that HemaMax may serve as a novel intervention for use as a frontline treatment to mitigate death due to radiation injury. First-in-Human, phase I studies are ongoing to assess the safety and pharmacokinetic and pharmcodynamic profiles of HemaMax, along with further efficacy studies in animals. The culmination of these human and animal studies will allow a determination of the predictive efficacious dose of HemaMax in humans under the Animal Rule, where efficacy is determined in animal models and safety is determined in humans.
